# Multi-site phosphorylation controls the neurogenic and myogenic activity of E47

**DOI:** 10.1016/j.bbrc.2019.02.045

**Published:** 2019-03-26

**Authors:** Laura J.A. Hardwick, John D. Davies, Anna Philpott

**Affiliations:** a Department of Oncology, University of Cambridge, Hutchison/MRC Research Centre, Cambridge Biomedical Campus, Cambridge, CB2 0XZ, UK; b Wellcome Trust/MRC Cambridge Stem Cell Institute, University of Cambridge, Tennis Court Road, Cambridge, CB2 1QR, UK; c Peterhouse, University of Cambridge, Trumpington Street, Cambridge, CB2 1RD, UK

**Keywords:** E47, *Xenopus*, Neurogenesis, Myogenesis, bHLH, Reprogramming, bHLH, basic-Helix-Loop-Helix, Cdk, Cyclin-dependent-kinase, ISH, In situ hybridisation, SP, Serine-Proline, TP, Threonine-Proline, WT, Wild-Type

## Abstract

The superfamily of basic-Helix-Loop-Helix (bHLH) transcription factors influence cell fate in all three embryonic germ layers, and the tissue-specific class II factors have received prominent attention for their potent ability to direct differentiation during development and in cellular reprogramming. The activity of many class II bHLH proteins driving differentiation, and the inhibitory class VI bHLH factor Hes1, is controlled by phosphorylation on multiple sites by Cyclin-dependent kinases (Cdks). As class II proteins are generally thought to be active through hetero-dimerisation with the ubiquitously expressed class I E proteins, regulation of class I transcription factors such as E47 may influence the activity of multiple tissue-specific bHLH proteins. Using differentiation of nerve and muscle in *Xenopus* frog embryos as a model system, we set out to explore whether with the ubiquitously expressed class I E protein E47 that hetero-dimerises with Class II bHLHs to control their activity, is also regulated by multi-site phosphorylation. We demonstrate that E47 can be readily phosphorylated by Cdks on multiple sites *in vitro*, while ectopically-expressed E47 exists in multiple phosphorylated forms in *Xenopus* embryos. Preventing multi-site phosphorylation using a phospho-mutant version of E47 enhances the neurogenic and myogenic activity of three different class II bHLH reprogramming factors, and also when E47 acts in hetero-dimerisation with endogenous proteins. Mechanistically, unlike phospho-regulation of class II bHLH factors, we find that preventing phosphorylation of E47 increases the amount of chromatin-bound E47 protein but without affecting its overall protein stability. Thus, multi-site phosphorylation is a conserved regulatory mechanism across the bHLH superfamily that can be manipulated to enhance cellular differentiation.

## Highlights

• E47 is phosphorylated in *Xenopus* embryos on up to 13 conserved SP/TP sites.
• *In vitro*, E47 protein can be phosphorylated by Cyclin-dependent-kinases.
• Under-phosphorylated E47 enhances activity of 3 bHLH reprogramming factors.
• Under-phosphorylated E47 enhances neuronal differentiation by hetero-dimerisation.
• E47 phospho-status alters chromatin binding but not overall protein stability.

## 1 Introduction

Basic-Helix-Loop-Helix (bHLH) proteins comprise a large superfamily of transcriptional regulators that are found in almost all eukaryotes, acting in the three embryonic germ layers as master regulators of cell fate [1]. Tissue specific class II bHLH proteins have received significant interest for their potent ability to direct differentiation, both during development and in cellular reprogramming. For example, MyoD was discovered for its ability to convert fibroblasts into myoblasts, and Ascl1 is a mainstay component of many neural reprogramming cocktails [2,3]. Accumulating evidence points to a conserved mechanism of multi-site phospho-regulation of these key class II bHLH reprogramming factors, including Ascl1, Ngn2, NeuroD4, MyoD and Ngn3: Their ability to drive differentiation is directly suppressed when Cyclin-dependent kinase (Cdk) activity is high, and preventing bHLH protein phosphorylation enhances lineage specific differentiation both *in vitro* and *in vivo* [[4], [5], [6], [7], [8]]. Recently, a similar mode of phospho-regulation has been shown to control the activity of Hes1, a class VI bHLH protein that suppresses differentiation, indicating that multi-site phosphorylation may play a broad role in controlling activity of bHLH transcriptional regulators [9].

Class II bHLH proteins are generally thought to be active through hetero-dimerisation with the ubiquitously expressed class I E proteins such as *Drosophila daughterless* and mammalian HEB, E2-2, E12 and E47 [1]. Functional hetero-dimerisation between their HLH domains has been shown to increase DNA binding affinity, alter DNA binding specificity and bring activation domains into functional proximity [10,11]. Thus, as ubiquitous hetero-dimerisation partners, E proteins have the ability to regulate the activity of multiple tissue-specific bHLH proteins.

Analogous to potential phosphorylation sites described in class II bHLH factors, E proteins also have multiple conserved Serine/Threonine Proline (SP/TP) sites, and phospho-regulation of these sites on a tissue-specific or global level has the potential to regulate differentiation across multiple lineages. To our knowledge, only one (S140) out of thirteen potential SP/TP sites in E47 has previously been ascribed a specific regulatory role in muscle differentiation [12]. We therefore set out to determine whether the activity of class I bHLH E protein E47 is controlled by multi-site phosphorylation on SP/TP sites, using differentiation of nerve and muscle in *Xenopus* frog embryos as a model system.

## 2 Materials and methods

### 2.1 Cloning

Wild-type (WT) mouse E47 (Genbank NM_011548.4) was cloned into pCS2 by In-fusion HD (Clonetec/Takara). 13T/S-A and 12T/S-A E47 were generated by QuikChange Site Directed Mutagenesis (Agilent). A single C terminal HA tag was added. WT mouse Ascl1, Ngn2 and MyoD, and 6S-A mouse Ascl1 have been described previously [4,5,7]. Tethered constructs were generated by PCR with the first component cloned into pCS2 between EcoR1 and Spe1 and the second component cloned into Cla1 and Xba1 to generate an in-frame fusion construct with flexible glycine-rich linker. All PCR primers available on request. Nucleotide and protein sequence alignments were conducted using ClustalW software [13].

### 2.2 *Xenopus laevis* embryo manipulation

All work has been carried out under UK Home Office Licence and in accordance with the UK Animals (Scientific Procedures) Act, 1986 and associated guidelines. A description of experiments using ARRIVE guidelines is provided in Ref. [14]. Acquisition of *X.laevis* embryos, preparation and injection of synthetic mRNA, and staging of embryos were conducted as described previously [6]. In situ hybridisation (ISH) was performed using dig-oxigenin-labelled anti-sense probes. Semi-quantitative scoring was conducted for gene expression on the injected side of the embryo relative to the uninjected side; grades were assigned: 0, no change in expression; +1, mild expansion within the endogenous domain only; +2, additional ectopic expression restricted to the dorsal ectoderm; +3, moderate but patchy ectopic expression spreading over the lateral ectoderm; +4, extensive ectopic expression over the lateral ectoderm in a homogenous pattern.

### 2.3 Quantitative real-time PCR

Whole embryo RNA was extracted, cDNAs prepared and qPCR conducted as described in Refs. [6,7].

### 2.4 Western blotting

*In vitro* kinase assay was conducted as described [8]. Protein extraction from whole embryos, lambda protein phosphatase treatment and assessment of cytoplasmic and chromatin-bound proteins was conducted as described [6], and all antibodies as used previously [6].

### 2.5 Statistical analysis

For western blotting, experiments were performed in independent triplicate with representative results shown. For qPCR data, mRNA expression was normalised to housekeeping gene *EF1α*, and mRNA levels in injected embryos were calculated relative to stage-matched uninjected controls. Mean values and the standard error of the mean (s.e.m.) were calculated from N independent experiments. Statistical significance was determined using a paired two-tailed Student's t-test with not significant = NS; (p < 0.05) = *; (p < 0.025) = **; (p < 0.0125) = ***. For ISH data, experiments were conducted in independent duplicate or triplicate and the N numbers refer to the range of total numbers of embryos in each injection category.

## 3 Results

### 3.1 E47 is phosphorylated *in vivo* on multiple Serine/Threonine-Proline sites

Multi-site phosphorylation of class II bHLH transcription factors by Cyclin-dependent kinases (Cdks) has been demonstrated to limit their ability to drive differentiation and reprogramming of cells in all three embryonic germ layers [[4], [5], [6], [7], [8]]. While limited evidence of phosphorylation-dependent regulation of E proteins is evident, for example Serine 140 in muscle [12], multi-site phospho-regulation of the E proteins E12 and E47 by Cdks has not been explored. E12 and E47 arise from alternative splicing of the E2A gene, varying only in a single exon that encodes different bHLH regions [15]. Thirteen Serine/Threonine-Proline (SP/TP) sites are present in the mouse E47 protein (analysed here), which may be targets for proline-directed kinases such as Cdks [[4], [5], [6], [7], [8]]. Nine of these potential phosphorylation sites lie outside any previously established functional domains of E47. Four sites are present in a lymphopoiesis-specific regulatory region EHD3 [16], and only Serine 140 (the second from N terminal SP in E47) has previously been described as a critical regulatory phosphorylation site for E47 in muscle [12]. We have used *Xenopus* as a flexible *in vivo* system to test whether the ability of E proteins to drive cellular differentiation is regulated by multi-site phosphorylation on Serine/Threonine-Proline sites during early embryogenesis.

**Fig. 1:**
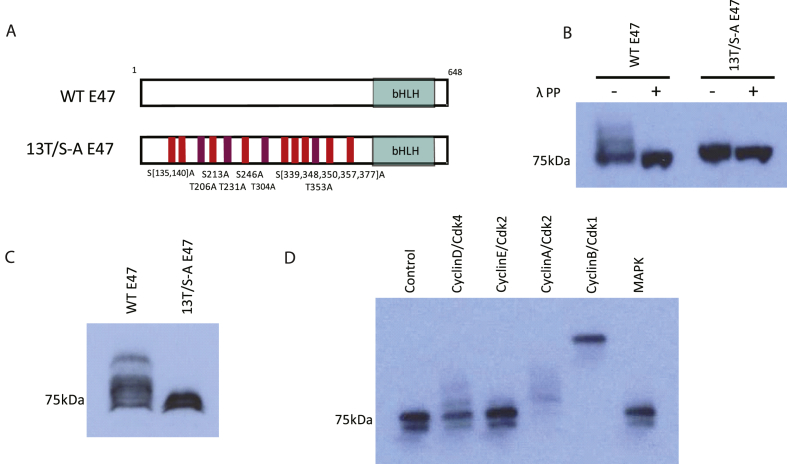
**E47 phospho-status is regulated *in vivo***. (A) Schematic representation of WT and 13T/S-A E47 constructs indicating the approximate location of the SP/TP sites mutated to AP. (B) Western blot with extracts of stage 11 embryos over-expressing 250 pg mRNA encoding WT or 13T/S-A E47, with or without λ-phosphatase treatment. (C) Phos-tag™ analysis of extracts from stage 11 embryos over-expressing WT or 13T/S-A E47. (D) *In vitro* kinase assay showing *in vitro* translated WT E47 protein after incubation with recombinant Cyclin/Cdks as labelled.

Firstly, we generated constructs encoding either WT E47 or 13T/S-A E47, where all of the potential SP/TP sites in E47 are mutated to Alanine-Proline (Fig. 1A). To determine whether E47 can be phosphorylated by proline-directed kinases as previously described for class II bHLH proteins [[4], [5], [6], [7], [8]], mRNA encoding WT or 13T/S-A E47 were injected into *Xenopus* embryos and protein extracts were prepared at stage 11. Western blotting (Fig. 1B) reveals that WT E47 migrates as multiple bands, consistent with post-translational modification, while enhanced migration on phosphatase treatment confirms that E47 is phosphorylated in embryos. In contrast, 13T/S-A E47 migrates more rapidly than WT as an apparently single broad band, and phosphatase treatment makes little difference to its migration. This indicates that the bulk of phosphorylation that affects E47 protein migration occurs on SP or TP sites that are absent in the phospho-mutant protein. To look more closely at potential modification of WT and 13T/S-A E47, we separated embryo extracts using Phos-tag™ gels that have previously been used to provide better separation of different phospho-forms of proteins [17]. On these gels (Fig. 1C), WT E47 from embryo extracts migrates as multiple distinct bands consistent with phosphorylation on a number of residues, while 13T/S-A E47 migrates as two closely-associated bands, likely indicating a modification on a non-SP/TP site. Thus, WT E47 is phosphorylated *in vivo* in developing *Xenopus* embryos on multiple SP/TP sites.

Class II bHLH transcription factors can be phosphorylated on SP/TP sites by Cdks [4,5,8]. To test whether E47 can also be phosphorylated by proline directed kinases such as Cdks or MAPK, we undertook *in vitro* recombinant kinase assays to phosphorylate E47 protein translated in reticulocyte lysate, and separating proteins on Phos-tag™ gel to more easily identify phosphorylated forms (Fig. 1D). Some modest slowed migration of E47 indicative of phosphorylation is observed on incubation with CyclinD/Cdk4, and more extensive modification is observed after incubation with CyclinA/Cdk2. However, the most dramatic retardation of E47 is observed after incubation with CyclinB/Cdk1, consistent with essentially all the E47 protein having undergone extensive phosphorylation. No significant change in migration is observed on incubation with CyclinE/Cdk2 or MAPK. Hence, E47 can be phosphorylated by Cdks, and there appears to be some selectivity for distinct Cyclin/Cdk combinations.

E proteins are traditionally viewed as the heterodimeric partners that work with class II bHLH transcription factors to drive neurogenesis or myogenesis [12,15]. We next tested whether phosphorylation of E47 affects its ability to drive or support differentiation in combination with the proneural factors Ascl1 or Ngn2, or with the myogenic factor MyoD. Forced expression of Ascl1 and Ngn2 can drive ectopic neuronal differentiation in the neural plate and lateral ectoderm of *Xenopus* embryos, while forced expression of MyoD expands myogenesis in the mesoderm [4,5,7]. Phosphorylation of S140 in E47 has previously been found to be essential for MyoD's ability to bring about transdifferentiation of fibroblasts into muscle [12], so an additional 12T/S-A (S140) E47 construct was made to restore the S140 site to compare any differences with the activity of 13T/S-A E47.

**Fig. 2:**
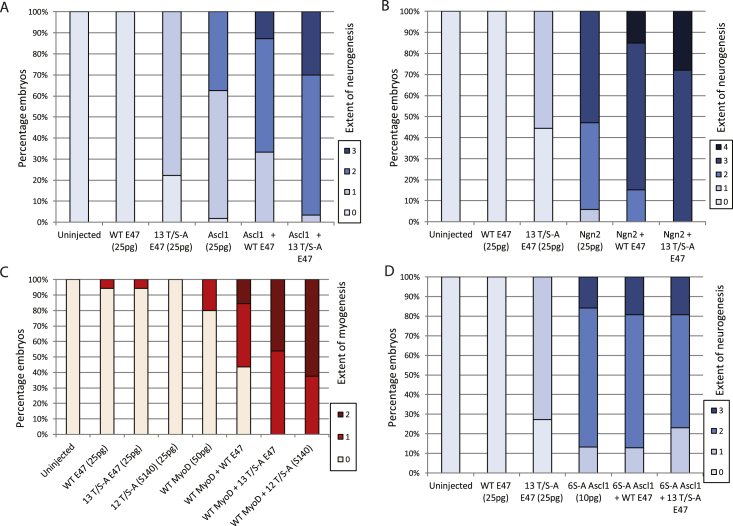
**Manipulation of E47 phospho-status enhances differentiation induced by WT bHLH reprogramming factors**. Embryos were injected as indicated and assayed at stage 18 by ISH for the extent of ectopic neurogenesis or myogenesis induced by WT Ascl1 (A) [N = 54–78], WT Ngn2 (B) [N = 25–40], WT MyoD (C) [N = 39–40], or phospho-mutant 6S-A Ascl1 (D) [N = 21–38]. Constructs and scoring system are described in the methods section.

WT and phospho-mutant E47 mRNAs were co-injected with WT Ascl1 (Fig. 2A), Ngn2 (Fig. 2B), or MyoD (Fig. 2C) into fertilised eggs, with embryos assayed at stage 18 for expression of N-tubulin as a marker of primary neurogenesis or muscle actin as a marker of myogenesis. Co-expression of WT E47 enhances induction of marker gene expression by all three class II proteins, and their neurogenic and myogenic activity is further elevated when 13T/S-A E47 is co-injected in place of WT E47. We see no significant difference between 13T/S-A E47 and 12T/S-A (S140) E47, suggesting that modification of S140 does not play a greater role in regulation of E47 than the combined activity of the other SP/TP sites.

Preventing phosphorylation of several class II bHLH proteins by mutating SP and TP sites results in their enhanced activity [[4], [5], [6], [7], [8]], and using phospho-mutant in place of wild-type proteins in reprogramming strategies results in improved conversion of mammalian fibroblasts into neurons [5]. We next tested whether expression of phospho-mutant E47 was further able to enhance neurogenic activity in combination with phospho-mutant (6S-A) Ascl1, when reprogramming *Xenopus* ectoderm into neurons (Fig. 2D). At this reduced dose of injected mRNA, ectopically expressed 6S-A Ascl1 induces a patchy ectopic increase in N-tubulin expression in approximately 90% of embryos, either in the presence of no ectopic E47, or after co-expression with WT E47. Ectopic neurogenesis is not significantly enhanced by substitution of 13T/S-A E47 in the place of WT E47, indicating that in this reprogramming setting there is no obvious added advantage of combining a phospho-mutant class II bHLH proneural factor with a phospho-mutant class 1 E protein.

### 3.2 The enhanced activity of phospho-mutant E47 depends on hetero-dimerisation

**Fig. 3:**
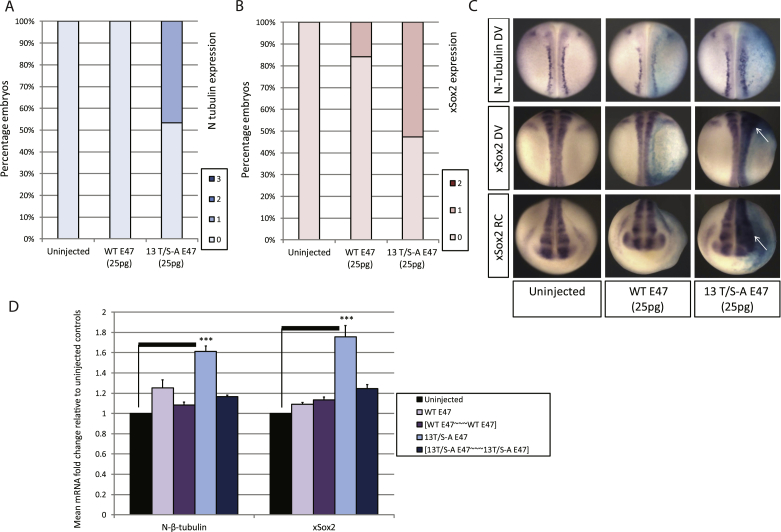
**E47 phospho-status influences endogenous neurogenesis in the neural plate**. Embryos were injected with mRNA as indicated and assayed by ISH at stage 18 for N-tubulin (A) [N = 15–18] or xSox2 (B) [N = 19–20] with representative images shown in (C), injected side to the right; DV, dorso-ventral; RC, rostro-caudal. (D) qPCR data from stage 18 embryos injected with molar equivalents of mRNA encoding E protein monomers or tethered homo-dimers [N = 3].

Class II bHLH proneural transcription factors are thought to act as hetero-dimers with E proteins to drive ectopic neurogenesis in *Xenopus*, while E proteins alone fail to upregulate neural genes in either whole embryos or explant assays [18]. To determine whether over-expression of hyperactive 13T/S-A E47 alone is sufficient to drive ectopic neurogenesis, we injected mRNA encoding WT or 13T/S-A E47 and compared neural-β-tubulin expression in stage 18 embryos. 13T/S-A but not WT E47 results in increased neurogenesis in the neural plate (Fig. 3A+C). To test whether this is due to expansion of neural progenitors in the neural plate, embryos were additionally assayed for expression of xSox2 (Fig. 3B+C). Sox2 is one of the earliest genes to be expressed in neural progenitor cells, and precedes the onset of expression of Ngn2, a class II bHLH factor that is the master regulator of the primary neuron cascade that results in neuronal differentiation [19]. While WT E47 injection has little effect on xSox2 expression, 13T/S-A E47 expands the rostral xSox2 domain in 50% of embryos. Therefore, even without additional class II bHLH proteins, phospho-mutant E47 but not WT E47 promotes endogenous neurogenesis *in vivo* through both an expansion of the progenitor domain and subsequent neuronal differentiation.

While E47 has been described to act through hetero-dimerisation in neurogenesis [15], E47 has an essential role as a homo-dimer in B cell development [20]. To investigate if the enhanced neurogenic activity of 13T/S-A E47 reflects phosphorylation-dependent changes in homo/hetero-dimer formation and activity, we made tethered constructs to test neurogenic activity of homo-dimers of WT and phospho-mutant E47. Molar equivalents of either monomeric or tethered WT or 13T/S-A E47 homo-dimers were over-expressed and embryos assayed by qPCR at stage 18 for N-tubulin and xSox2 expression (Fig. 3D). The enhanced neurogenic activity of the phospho-mutant E47 monomer when injected alone is not shared with the forced homo-dimer composed of two tethered 13T/S-A E47 proteins, and tethered WT E47 is also inactive. This indicates that the enhanced activity of 13T/S-A E47 in induction of neurogenesis is dependent on its hetero-dimerisation with other transcription factor partners.

### 3.3 Preventing phosphorylation of E47 enhances its association with chromatin

**Fig. 4:**
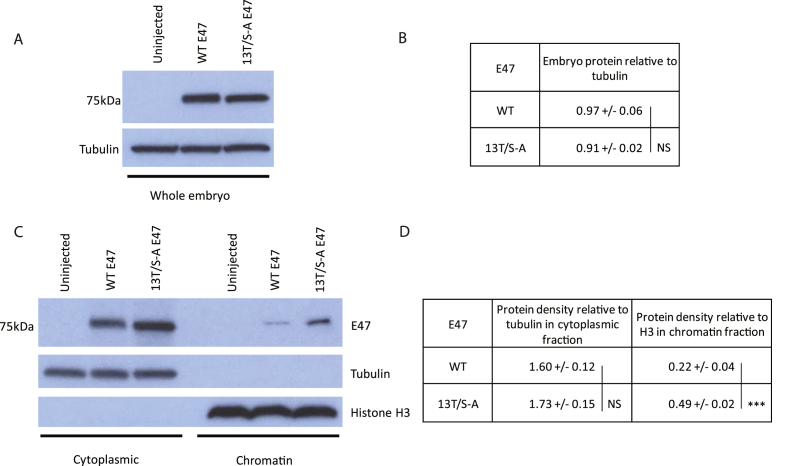
**Phospho-mutant E47 has enhanced chromatin association relative to WT E47**. Embryos over-expressing WT and 13T/S-A E47 were analysed by western blot of whole embryo extracts at stage 11 (A) or cytoplasmic and chromatin fractions at stage 13 (C) with E47 protein density calculated relative to loading controls in (B) and (D) respectively. Significance determined using a paired two-tailed Student's t-test; NS = not significant; *** = p < 0.0125.

Finally, we explored the mechanisms by which phosphorylation might regulate the activity of E47. Preventing multi-site phosphorylation of class II bHLH proteins has been shown to increase protein stability [[6], [7], [8]]. To determine whether ectopically expressed phospho-mutant E47 accumulates to a higher level than WT protein, whole embryo extracts were prepared from stage 11 embryos injected with mRNA encoding WT or 13T/S-A E47. When the density of the E47 protein band is measured relative to tubulin loading control, we find no significant differences in protein accumulation of 13T/S-A E47 compared to WT E47 (Fig. 4A+B). Thus, phosphorylation appears to have no significant effect on E47 protein stability.

We have also previously seen that preventing multi-site phosphorylation can enhance chromatin binding of class II bHLH proteins [[4], [5], [6], [7]]. To determine whether this is also the case for E47 we undertook subcellular fractionation of *Xenopus* embryos followed by western blotting to detect E47 protein in cytoplasm and bound to chromatin (Fig. 4C+D). Interestingly, while there is no significant difference in E47 accumulation in the cytoplasm, we detect approximately twice as much 13T/S-A E47 compared to WT E47 in the chromatin fraction. Therefore, preventing phosphorylation of E47 enhances its ability to bind to chromatin and this is likely to underpin its enhanced ability to drive differentiation.

## 4 Discussion

We find that E47 is phosphorylated in *Xenopus* embryos on up to 13 conserved SP/TP sites and mutation of these sites enhances both endogenous neurogenesis and differentiation induced by ectopic class II bHLH reprogramming factors. E47 is phosphorylated *in vitro* by CyclinA/Cdk2 and CyclinB/Cdk1, suggesting potential regulation during the G2/M phase of the cell cycle, although we note that there is no measure of relative kinase activity against another independent substrate making it hard to draw firm conclusions about kinase specificity from this experiment. We would however note that a similar kinase assay using the class II bHLH proneural protein Neurogenin3 demonstrates phosphorylation by all four Cyclin/Cdk combinations [8], consistent with the possibility that different bHLH proteins may be targeted to different extents by distinct Cyclin/Cdk complexes.

Class II bHLH transcription factors typically have short half-lives of around 20 min, while preventing their multi-site phosphorylation results in significant protein stabilisation [8,21]. In contrast, we do not see an increase in E47 half-life when its phosphorylation is prevented in *Xenopus* embryos. We note that *Drosophila* homologue *daughterless* has a long half-life of over 4 h during neurogenesis [22], suggesting that protein turn-over rate of class I bHLH proteins may not be as tightly regulated as that of class II bHLH factors. We do however see that under-phosphorylated E47 has greater chromatin association compared to WT E47. This is likely to explain its enhanced ability to expand the neural progenitor pool and drive enhanced neurogenesis in the neural plate of *Xenopus* embryos; an effect that requires hetero-dimerisation with endogenous factors. Thus, multi-site phosphorylation controls the activity of classes I, II and VI bHLH transcription factors [[4], [5], [6], [7], [8], [9]], representing a common mechanism that allows transcriptional activity to be responsive to cell signalling via activation of Cdks and potentially other kinases.

In the field of mammalian cellular reprogramming, class II bHLH proteins such as Ascl1 are routinely utilised for their potent ability to direct cell-type specific differentiation, but advances in our understanding of the control of the reprogramming process is needed to improve both the conversion efficiency and maturity of induced cells. Phospho-mutant 6S-A Ascl1 shows an enhanced ability to convert mammalian fibroblasts into mature neurons compared to the wild-type protein, and generates more ectopic neurons in *Xenopus* ectoderm compared to WT Ascl1 [5,23]. However, we do not find further enhancement of ectopic neurogenesis in *Xenopus* by supplying extra WT or phospho-mutant E47 along with 6S-A Ascl1 (Fig. 2D). 6S-A Ascl1 already has increased protein stability and DNA binding affinity compared to WT Ascl1 [5], indicating that de-phosphorylation of both components of the bHLH dimeric complex is not required for maximal activity.

Taken together, our data suggest that cycle lengthening and exit during development may result in de-phosphorylation of both class I and class II components of the hetero-dimeric bHLH complex, and both components may contribute to enhancement of differentiation. Furthermore, our data suggest that using forms of class II or class I bHLH proteins that cannot be phosphorylated by Cdks may enhance *in vitro* cell reprogramming.
